# Monocyte infiltration rather than microglia proliferation dominates the early immune response to rapid photoreceptor degeneration

**DOI:** 10.1186/s12974-018-1365-4

**Published:** 2018-12-15

**Authors:** Sarah J. Karlen, Eric B. Miller, Xinlei Wang, Emily S. Levine, Robert J. Zawadzki, Marie E. Burns

**Affiliations:** 1 Department of Cell Biology and Human Anatomy, University of California, Davis, 1 Shields Avenue, Davis, CA 95616 USA; 2 Center for Neuroscience, University of California, Davis, 1544 Newton Court, Davis, CA 95618 USA; 3 Department of Ophthalmology & Vision Science, University of California, Davis, 1 Shields Avenue, Davis, CA 95616 USA

**Keywords:** Neuroinflammation, Retina, Macrophage, Monocyte, Myeloid cells, Microglia, Photoreceptor, Rod, Cone

## Abstract

**Background:**

Activation of resident microglia accompanies every known form of neurodegeneration, but the involvement of peripheral monocytes that extravasate and rapidly transform into microglia-like macrophages within the central nervous system during degeneration is far less clear.

**Methods:**

Using a combination of in vivo ocular imaging, flow cytometry, and immunohistochemistry, we investigated the response of infiltrating cells in a light-inducible mouse model of photoreceptor degeneration.

**Results:**

Within 24 h, resident microglia became activated and began migrating to the site of degeneration. Retinal expression of CCL2 increased just prior to a transient period of CCR2^+^ cell extravasation from the retinal vasculature. Proliferation of microglia and monocytes occurred concurrently; however, there was no indication of proliferation in either population until 72–96 h after neurodegeneration began. Eliminating CCL2-CCR2 signaling blocked monocyte recruitment, but did not alter the extent of retinal degeneration.

**Conclusions:**

These results demonstrate that the immune response to photoreceptor degeneration includes both resident microglia and monocytes, even at very early times. Surprisingly, preventing monocyte infiltration did not block neurodegeneration, suggesting that in this model, degeneration is limited by cell clearance from other phagocytes or by the timing of intrinsic cell death programs. These results show monocyte involvement is not limited to disease states that overwhelm or deplete the resident microglial population and that interventions focused on modulating the peripheral immune system are not universally beneficial for staving off degeneration.

**Electronic supplementary material:**

The online version of this article (10.1186/s12974-018-1365-4) contains supplementary material, which is available to authorized users.

## Key points

- Transient infiltration of Cd11b^+^ CD45^+^ Ly6C^+^ CCR2^+^ monocytes from retinal vessels occurs within 24 h of the onset of photoreceptor stress.
- Monocyte recruitment is preceded by increased retinal CCL2 expression.
- Blocking the CCL2-CCR2 signaling pathway prevents monocyte recruitment, but does not alter the overall rate of degeneration.
- Both microglia and monocyte proliferation occur concurrently, but not until several days after the onset of neurodegeneration, when the rate of cell death has slowed.

## Background

Microglia are the resident immune cells of the central nervous system [1–5]. In healthy retina, microglia are confined to the synaptic and ganglion cell layers, where they survey their surrounding environment with dynamic ramifications to maintain tissue homeostasis and normal synaptic function [6]. During neurodegeneration, local changes in chemokines, purines, and the ionic milieu cause microglia to lose their ramifications and become amoeboid, a cellular state capable of rapid migration and greater phagocytic capacity [7]. Bone marrow-derived monocytes can also be recruited from the systemic circulation to further escalate the inflammatory response, often when the microglia population becomes depleted ([8] for review). Because infiltrating monocytes differentiate into macrophages that become difficult to distinguish from resident immune cells [9], determining the roles of these populations in the progression of degeneration has been difficult.

One method commonly used to separate resident microglia from infiltrating monocytes has been bone marrow transplant studies [9, 10]. A drawback to this method is that the radiation that kills myeloid cells can kill microglia as well, leading to the establishment of non-microglia macrophages in the retina that out-populate bona fide microglia and obscure the natural dynamics during neurodegeneration [11, 12]. A second approach has been to deplete circulating monocytes with clodronate liposomes, which in some models delays degeneration [13, 14], suggesting that infiltrating cells are primarily harmful. However, because the characteristics of both resident and infiltrating cells can change over time, a deeper understanding of their respective roles requires disambiguating their molecular phenotypes and correlating individual phenotypes with cellular behaviors during the onset and progression of neurodegenerative disease ([15–17] for review).

The Arrestin-1 knockout mouse (*Arr1*^*−/−*^ *aka Sag*^*−/−*^) [18] allows the natural immune response to cell-autonomous neurodegeneration to be readily controlled and studied over time. Arrestin-1, also known as S-antigen or visual arrestin, is only expressed in rod and cone photoreceptors where it is responsible for quenching the activity of light-activated rhodopsin in the phototransduction cascade [19]. In mice lacking Arrestin-1, phototransduction signaling in retinal rods is greatly prolonged and results in light-induced photoreceptor degeneration from within an otherwise fully developed, normal retina [18, 20–23]. As a result, exposing dark-reared animals to normal lab lighting (25–200 lx) induces widespread microglial activation and a dramatic increase in Iba1^+^ cell numbers in the retinas of *Arr1*^*−/−*^ mice [24]. Since Iba1 is expressed in both microglia and macrophages, this observation led us to examine whether the increase in Iba1^+^ cell number is primarily due to microglial proliferation or to monocyte infiltration in order to assess the roles that these populations play during degeneration.

Since Arrestin-1 is only expressed in rod and cone photoreceptors, it is an ideal model for studying the inflammatory response to single cell-specific degeneration within the central nervous system. Unlike models where the experimental manipulation itself can contribute to an immune response, the effects measured in this model are caused directly by the degeneration of a single neuronal cell type. Thus, the conclusions reached can be extrapolated to models of disease that are also due to neuron-specific degeneration. In the eye, these types of diseases often fall under the broad category of hereditary retinopathy (e.g., retinitis pigmentosa), which can affect photoreceptors or retinal pigment epithelium cells and represents a significant cause of blindness in humans ([25] for review).

Here, we show that in the *Arr1*^*−/−*^ retina, photoreceptor stress and degeneration is accompanied by a rapid infiltration of monocytes from the retinal vasculature. Recruitment occurs subsequent to increased CCL2 in the retina and several days before the resident microglial cells show any change in population size. Eliminating CCL2-CCR2 signaling blocked monocyte infiltration but did not alter the extent of degeneration. These results demonstrate that the immune response to neurodegeneration includes resident and infiltrated cells, even at very early times, and that monocyte involvement is not limited to disease states that overwhelm or deplete the resident microglial population and does not always hasten degeneration.

## Methods

### Animals

Mice were cared for and handled in accordance with the National Institutes of Health guidelines for the care and use of experimental animals and under approved protocols by the Institutional Animal Care and Use Committee of the University of California, Davis. All mice, including *Arr1*^*−/−*^ [18] and wildtype C57BL/6J mice (Jackson Laboratories; Jax 000664), were born and raised in 24-h darkness prior to light exposure. *Arr1*^*−/−*^ mice were crossed with CCL2-RFP^lox/lox^ mice (Jax 016849) and PDGFRa-CRE mice (Jax 013148), creating *Arr1*^*−/−*^ *CCL2-RFP*^*lox/lox*^ *PDGFRa-CRE*^*−*^ and *Arr1*^*−/−*^ *CCL2-RFP*^*lox/lox*^ *PDGFRa-CRE*^*+*^ mice that were sensitive to light, expressed a modified CCL2 gene tagged with a red fluorescent protein (mcherry), and, in Cre^+^ mice, were deficient in Müller cell CCL2 expression. *Arr1*^*−/*−^ mice were also crossed with *CCR2*^*rfp/rfp*^ mice (Jax 017586) to generate *Arr1*^*−/*−^ *CCR2*^*+/rfp*^ and *Arr1*^*−/*−^ *CCR2*^*rfp/rfp*^ animals used for in vivo imaging and flow cytometry.

To initiate photoreceptor degeneration, adult mice between 2 and 5 months of age were exposed to homogeneous, continuous light of 75–200 lx for up to 240 h. Both males and females were used; mice were screened for the rd8 mutation [26] and found to be lacking the mutation. Mice were euthanized by CO_2_ narcosis.

### Immunohistochemistry

Following euthanasia, eyecups were dissected and fixed with 4% paraformaldehyde (PFA) in phosphate-buffered saline (PBS). Eyecups were stored in PBS at 4 °C at least overnight before connective tissue was trimmed off prior to sectioning. Eyecups were oriented to keep track of superior-inferior polarity while embedded in 4% low-melting agarose, then sectioned at 150 μm. Sections were blocked, incubated in primary antibodies overnight at 4 °C, washed in PBS, then incubated in secondary antibodies with DAPI (R37606, Invitrogen) for 1.5–2 h at room temperature. After an additional wash in PBS, sections were mounted onto slides in Prolong Diamond Antifade Mountant with DAPI (P36971, Invitrogen). Slides were visualized with a Nikon Ti-E A1 multiphoton imaging system using a × 40-water immersion objective. Thirty-micrometer z-stacks and high-resolution images were sampled using NIS-Elements Microscope Imaging Software (Nikon). For IHC quantification, oblique sections were excluded from analysis. For each time point, between 2 and 9 sections from 2 to 3 mice per strain were quantified. Counts were standardized based on retinal area.

The following primary antibodies were used for IHC: rabbit anti-Iba1 (1:1000, 019-19741, Wako), rat anti-CD11b (1:300, ab24874, Abcam), mouse anti-Glutamine Synthetase (GS, 1:1000, MAB302, EMD Millipore), goat anti-CCL2/JE/MCP-1 (1:300, AF-479-NA, R&D Systems), and Alexa Fluor 647 anti-mouse/human CD11b (1:300, 101218, Biolegend). Secondary antibodies were diluted 1:300 and purchased from Invitrogen. Immunosera were diluted in a buffer of PBS with 0.5% bovine serum albumin and 0.5% Triton X-100.

### In vivo imaging and analysis

For retinal imaging, the mice were initially anesthetized with 4% isoflurane, sustained at 2–2.5%, and their body temperature maintained by heating pad (37 °C) attached to micropositioner (Phoenix Research Labs) that allowed rotational and translational adjustment for eye alignment. The pupils were dilated and cyclopleged with tropicamide and phenylephrine, and the corneal surface wetted with hypromellose gel (GenTeal Tears Severe, Alcon) and covered with a 0-Dpt. contact lens (Unicon Corporation).

A custom-built multimodal scanning laser ophthalmoscopy (SLO) and optical coherence tomography (OCT) system were used for all in vivo imaging [27]. Both SLO and OCT widefield images were collected over 51° visual angle (2.2 mm). OCT imaging (100 B-scans and 2000 A-scans/B-scans) was used to monitor the retina during degeneration. OCT B-scan images were collected before light exposure (under dim red light used for alignment) and then after 24, 48, 72, 96, and 240 h of light exposure in the same animals. The OCT subsystem collects A-scans (single tomographic axial samples) at 100 kHz, with an axial resolution ~ 2 μm, while the SLO and OCT subsystems have *x*-, *y*-analog resolutions of ~ 3.5 μm, respectively. The OCT light source was centered at 860 nm with a 132-nm bandwidth (superluminescent diode, Broadlighter T-860-HP; Superlum, Carrigtwohill, Cork, Ireland). To measure the thickness of retinal layers, all A-scan intensity profiles were averaged from the flattened OCT volume and evaluated using the choroid, INL, and RGC as landmarks.

For SLO images, multiple frames were averaged from wide-field images to increase visibility of retinal structures. To image RFP^+^ cells in the CCR2-RFP^+/rfp^ mouse, SLO images were acquired using 560-nm excitation light (OBIS Laser, Coherent); back-reflected (retina morphology) and fluorescent light (RFP expressing cells) was collected using dichroic splitter (Semrock). To observe movement of RFP^+^ cells in the vasculature and retina, we restricted the SLO acquisition field to 10–13° visual angle (457–556 μm) which resulted in increased pixel sampling density. The SLO frames were captured at 1.5 frames per second.

### EdU pulse

Mice were given a single intraperitoneal (IP) injection of EdU (5-ethynyl-2′-deoxyuridine) at 100 mg/kg of 5 mg/ml concentration at light onset (time 0) and exposed to light for 0, 24, or 48 h. After euthanasia, tissues were dissected and EdU was identified using the Click-iT EdU Alexa Fluor 647 Imaging Kit for IHC (C10640, Invitrogen) or the Click-iT EdU Pacific Blue Flow Cytometry Assay Kit for flow cytometry (C10418, Invitrogen) according to the manufacturer’s instructions. For flow cytometry, each retina was considered a single sample, and up to 2.5 million events were collected; three blood samples, six retinas, and six choroids were used per time point, and collection was balanced such that at least one animal for each time point was quantified during each experimental run.

### Flow cytometry

Flow cytometry was performed using a modified protocol based on O’Koren et al. [9]. Briefly, a tail-vein injection of either PE anti-mouse CD45 antibody (103106, BioLegend) or APC anti-mouse CD45 antibody (103112, BioLegend) was administered 5 min prior to euthanasia in order to label immune cells trapped in the circulating vasculature. Mice were euthanized by CO_2_ narcosis, and the retinas and RPE-choroid complex were digested in 1 mL of Hibernate medium (HBSS) (10-547F, Lonza) supplemented with 5% fetal bovine serum (FBS), 10 mM HEPES, 0.7 mg/ml calcium chloride, 1.5 mg/ml of Collagenase A (10103586001, Roche), and 0.1 mg/ml DNase I (10104159001, Roche). The tissue was digested at 37 °C for 15–20 min, filtered through a 70-μm cell strainer (25–276, Genesee Scientific), and resuspended in PBS. Cells were stained for viability (Zombie NIR Fixable Viability Kit, 423106, BioLegend) for 15–30 min; blocked with Fc block 1 ul/sample (14-0161-86, eBioscience), 5% normal rat serum, and 5% normal mouse serum for 5 min; then incubated with antibodies (1:100) at 37 °C for 60 min. Antibody combinations varied depending on the experiment: APC anti-mouse CD45 (103112, BioLegend), Brilliant Violet (BV) 605 anti-mouse/human CD11b (101257, BioLegend), BV 421 anti-mouse CX3CR1 (149023, BioLegend), BV 421 anti-mouse CD45 (103134, BioLegend), Alexa Fluor 488 anti-mouse Ly-6C (128022, BioLegend), PE/Cy7 anti-mouse CD192 (CCR2; 150612, BioLegend), BV 711 anti-mouse CX3CR1 (149031, BioLegend), and BV 711 anti-mouse I-A/I-E (107643, BioLegend). Antibody master mix was made in Brilliant Stain Buffer (563794, BD Biosciences). Cell suspensions were washed in 0.5% bovine serum albumin with 1:50 EDTA, and resuspended in Cell Staining Buffer (420201, BioLegend) with 0.5% PFA.

Bead controls were created using AbC Total Antibody Compensation Bead Kit (A10497, Invitrogen) and ArC Amine Reactive Compensation Bead Kit (A10628, Invitrogen). Data were acquired on a BD LSRII flow cytometer (BD Biosciences) and analyzed with FlowJo software (Tree Star). Each retina was considered a single sample, and all events, up to 2.5 million, were collected for each sample; a total of 3–5 blood samples, 6–9 retinas, and 6–9 choroids were collected per time point. Data collection was balanced such that at least one animal for each time point was quantified during each experimental run. The gating strategy was based on references [9, 28–32].

### Statistical analysis

Statistical analyses were performed using R (R core team, 2017, version 3.4.1). Results are presented as mean ± SEM. A multiple-group comparison was performed using a one-way ANOVA followed by Tukey Honest Significant Differences post hoc test for all statistical analyses in the study. The significance level was set at *p* < 0.05 (shown as * in figures), *p* < 0.01 (** in figures) and *p* < 0.001 (*** in figures).

### Multiple cytokine arrays

The relative levels of 40 different cytokines were determined using mouse cytokine antibody array (Panel A, R&D System), according to the manufacturer’s protocol. Retinas were homogenized in PBS with protease inhibitors (cOmplete, mini, EDTA-free cocktail tablets, Roche). Samples were centrifuged at × 10,000*g* for 5 min to remove cellular debris, and Triton X-100 was added to a final concentration of 1%. Protein concentrations were quantified using total protein assay (Pierce 660 nm Protein Assay Kit, Thermo Fisher Scientific). Pre-mixed sample/antibody cocktails were incubated with blocked membranes overnight at 4 °C on a rocking platform shaker. After 3 washes, membranes were incubated with 800CW streptavidin (IRDye, LI-COR) for 30 min and washed 3 times before being scanned on the LI-COR odyssey imaging system. Data were analyzed with image studio lite 4.0 software (LI-COR). The average fluorescent intensity of each cytokine was quantified for each retinal sample; the light-dependent changes in cytokine expression were determined by dividing the light-exposed values by those of the dark-reared samples processed in parallel from the same genotypes (WT and *Arr1*^*−/−*^) and performed in triplicate.

### ELISAs

Retinas were homogenized in 100 μl of PBS with protease inhibitors (cOmplete, mini, EDTA-free cocktail tablets, Roche) using sonication, then centrifuged at 14,000 rpm at 4 °C for 15 min to remove cellular debris. Protein concentrations were quantified using total protein assay (Pierce 660 nm Protein Assay Kit, Thermo Fisher Scientific). CCL2 levels were determined using the quantitative sandwich enzyme immunoassay technique (MJE00, R&D Systems), according to the manufacturer’s instructions. Both eyes from each animal were combined and tested as a single sample; 3–5 mice were run for each time point.

### CCL2 neutralizing antibody injections

After 12 h of light exposure, mice were anesthetized with 2–4% isoflurane mixed with oxygen. Pupils were dilated topically with 1% tropicamide and 2.5% phenylephrine ophthalmic solution. A small incision was made with the bevel of a 33-G needle, and a 2.5-μl Hamilton syringe was used to inject 1.5–2 μl of solution into the vitreous. Mice received either saline or 0.2 mg/ml goat anti-CCL2/JE/MCP-1 (1:300, AF-479-NA, R&D Systems) in sterile saline.

## Results

### In vivo imaging reveals transient period of monocyte extravasation

**Fig. 1:**
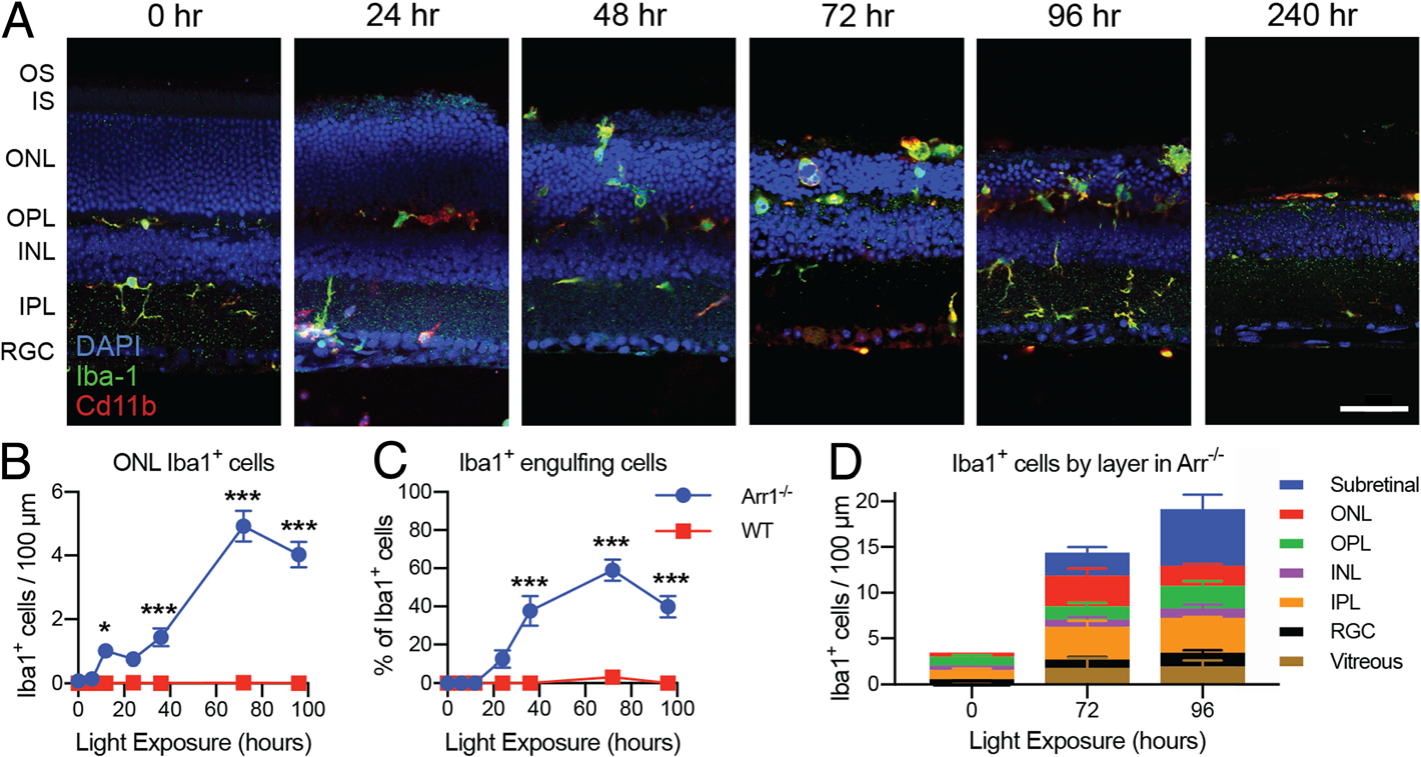
*Timecourse of microglia activation and photoreceptor clearance.* **a** IHC of retinal sections of dark-reared (0 h) and light-exposed *Arr1*^*−/−*^ mice (24–240 h). Light induced widespread migration of Iba1^+^ cells (green) into the outer retina. By 72 h, the outer retina (OS, IS, ONL) was markedly thinned and by 240 h had nearly vanished. **b**–**d** Quantification of Iba1^+^ cells in IHC sections. **b** The number of Iba1^+^ cells in the ONL per 100 μm increased significantly in two phases after light onset in *Arr1*^*−/*−^ but did not change in dark-reared wild type (WT) controls. **c** The percent of Iba1^+^ cells actively engulfing photoreceptors increased significantly at 36 h in Arr1^−/−^ and remained elevated through 96 h; there was no change in WT. Data in B and C re-analyzed from Levine et al., 2014. **d** In Arr1^−/−^ mice, the majority of Iba1^+^ cells in dark-reared animals (0 h) were located in the plexiform layers, but after 72–96 h of light exposure, Iba1^+^ cells were also observed in the vitreous, RGC, ONL, and subretinal layers. Note that the discrepancy in quantification between 1B and 1D is due to the inclusion of subretinal and vitreal Iba1^+^ cells in 1D, which were not counted in the first study. Vitreal Cd11b^+^ cells may include a population of hyalocytes. Scale bar is 50 μm in A. OS photoreceptor outer segment layer, IS photoreceptor inner segment layer, ONL outer nuclear layer, OPL outer plexiform layer, INL inner nuclear layer, IPL inner plexiform layer, RGC retinal ganglion cell layer. *p < 0.05, **p < 0.01, ***p < 0.001; error bars are SEM, *n* = 7–18 locations per time point (locations selected from 3 to 9 sections across 2–3 mice per strain per time)

In dark-reared mice lacking Arrestin-1, phototransduction signaling in retinal rods is greatly prolonged and results in light-induced photoreceptor degeneration from within an otherwise fully developed, normal retina ([18, 21]; Fig. 1a). The abrupt onset of widespread photoreceptor degeneration activates retinal microglia (Iba1, green; Fig. 1a), which migrate into the photoreceptor layers of the retina along a well-defined time course initiated by the onset of light (Fig. 1a, b) (see also [24]). By 36 h, a significant fraction of Iba1^+^ cells had begun to engulf and presumably phagocytose photoreceptor cell bodies in the outer nuclear layer (ONL; Fig. 1c). After 96 h of light exposure, ~ 40% of the Iba1^+^ cells were located in the ONL and subretinal space (Fig. 1d, red and blue), in stark contrast to their normal position in the synaptic layers of dark-reared wildtype retinas, or *Arr1*^*−/−*^ retinas prior to light exposure (Fig. 1a, b). Wildtype mice processed in parallel did not show ONL thinning or changes in Iba1^+^ cells at any time point (Fig. 1b) (see also [24]).

The number of Iba1^+^ cells in the ONL appeared to increase in two distinct phases, an initial increase within 24 h and a slower rise that peaked at 72–96 h, possibly reflecting both the migration of resident cells and the infiltration of monocytes, both of which can express Iba1. Indeed, close inspection of retinal sections co-stained for Cd11b revealed small round cells adhering along the vitreal surface of *Arr1*^*−/−*^ retinas typical of monocyte morphology (Additional file 1: Figure S1A compare 0 to 24 h). It is worth noting that Cd11b^+^ cells with a more pleiotropic morphology could be hyalocytes [33], the resident macrophage population of the vitreous, rather than monocytes. The infiltrates appeared most abundant at the optic nerve head and surrounding peripapillary region (Additional file 1: Figure S1B). The sudden appearance of infiltrating cells at the vitreoretinal surface was also readily observed in vivo using optical coherence tomography (OCT). Small, punctate light scattering signals appeared in the vitreous after 24 h of light exposure in *Arr1*^*−/−*^ mice, but not wildtype controls (Additional file 1: Figure S1C, arrows), consistent with an influx of immune cells from vessels at the retinal surface.

**Fig. 2:**
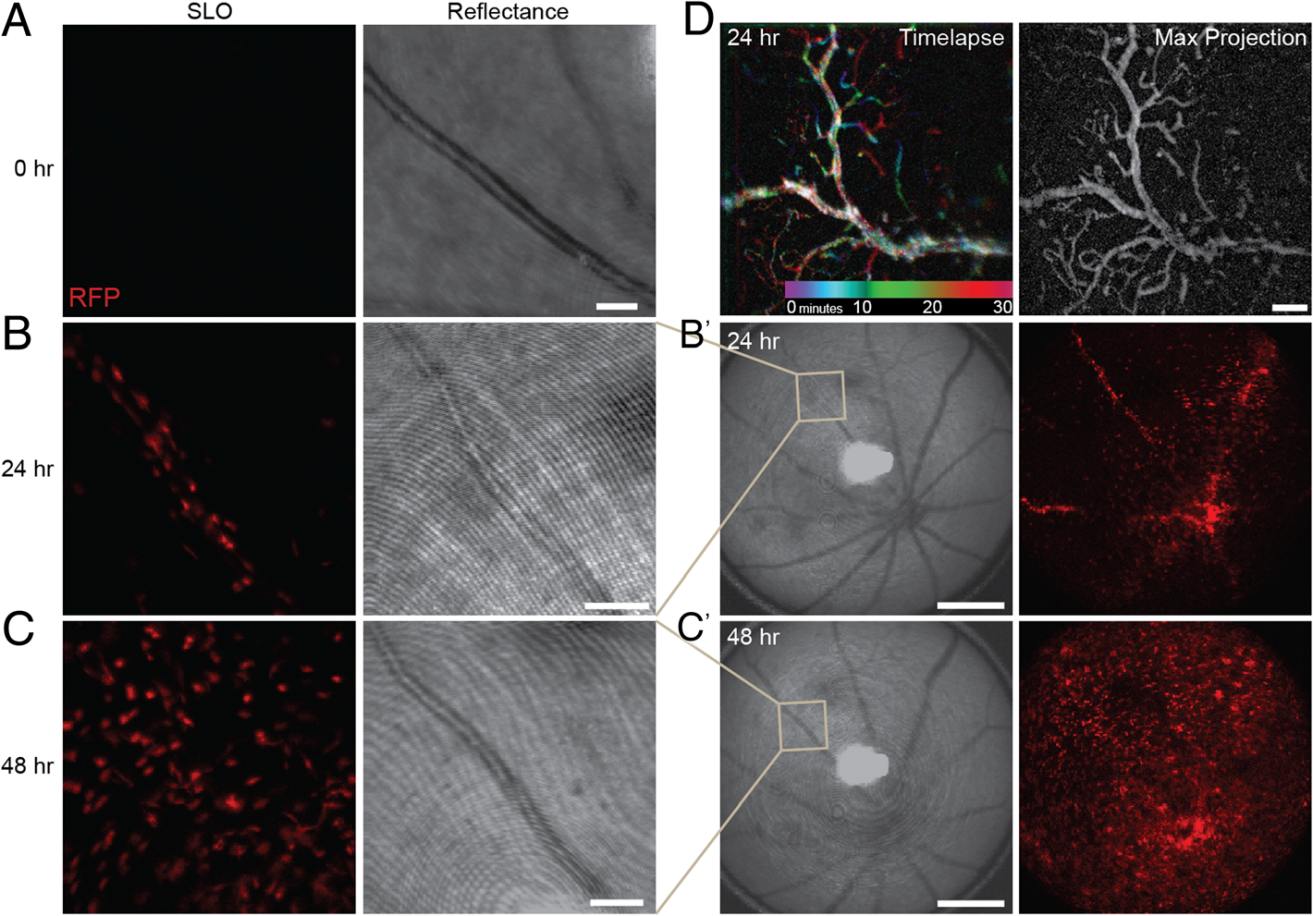
In vivo *imaging of monocyte infiltration through retinal vasculature during early neurodegeneration.* In vivo retinal images of *Arr1*^*−/−*^ *CCR2*^*+/rfp*^ mice before and during photoreceptor degeneration. The fluorescence images (SLO) showed only fleeting RFP^+^ signal prior to degeneration **(a**), but in the same vessel 24 h later, RFP^+^ cells studded the interior (**b**) and could also be seen in other large caliber vessels and within the parenchyma of the central retinal (**b**′). By 48 h, RFP^+^ cells had escaped the vasculature and appeared throughout the retina (**c**, **c**′). The bright field (reflectance) images taken concurrently with the fluorescence (SLO) show the position of the blood vessel in the same animal over time. SLO images are frames from Additional file 2: Video 0 h; Additional file 3: Video 24 h; Additional file 5: Video 48 h. (**d**) Color-coded representation of Additional file 4: Video ext 24 h (different *Arr1*^*−/−*^ *CCR2*^*+/rfp*^ animal, 24 h), where each pixel was colorized based on the time epoch of the video in which RFP^+^ cells appeared. The largest diameter vessel is largely white because the corresponding pixels were RFP^+^ throughout the entire session due to persistent RFP^+^ cell adherence to the vessel walls. Notice transient epochs of cell adherence to finer capillaries as revealed by streaks of blue, green, red, etc. The grayscale projection of maximal pixel intensities over time (right) outlines the vascular network in greater detail. Scale bars are 100 μm in **a**, **b**, **c**, **d**, and 500 μm in **b**′, **c**′


To further examine infiltration, mice expressing RFP behind the CCR2 promoter were bred into the *Arr1*^*−/−*^ background (*Arr1*^*−/−*^ *CCR2*^*+/rfp*^) and used to visualize monocytes within the retinal vessels in vivo with scanning laser ophthalmoscopy (SLO). Prior to the onset of degeneration (0 h), RFP^+^ cells were only fleetingly visible in the retinal vasculature (Fig. 2a; Additional file 2: Video 0 h). After 24 h of light exposure, RFP^+^ cells were evident adhered to and rolling along the vessel walls (Fig. 2b, b’; Additional file 3: Video 24 h). Extended real-time in vivo imaging showed RFP^+^ monocytes sometimes paused before detaching and re-entering the circulation, other times they rolled to a stop and extravasated through the vessel walls of the deep retinal capillaries (Fig. 2d; Additional file 4: Video ext 24 h). By 48 h, the retinal area outside the vessels was studded with round RFP^+^ cells that had extravasated and appeared to be mobile and migratory; many fewer cells were visible within the vessels than 24 h earlier (Fig. 2c, c’; Additional file 5: Video 48 h). Together, these data show that CCR2^+^ monocytes transiently adhere to the endothelial cells and extravasate from retinal vessels into the capillary plexus within the first 48 h of neurodegeneration.

**Additional file 2:** Video 0 h. In vivo imaging of monocyte infiltration at 0 h. The fluorescence image (SLO) showed only fleeting RFP^+^ signal prior to degeneration (0 h), indicating that under normal conditions, RFP^+^ cells course through the vasculature at a rapid rate. Cells were pseudocolored red; scale bar given in Fig. 2a. (AVI 4330 kb)



**Additional file 3:** Video 24 h. In vivo imaging of monocyte infiltration at 24 h. By 24 h, RFP^+^ cells studded the interior of the retinal vessel, adhering to the retinal walls and slowly rolling along the interior surface. Recorded from the same vessel shown at 0 h, which had previously shown only fleeting RFP^+^ cells. Cells were pseudocolored red; scale bar given in Fig. 2b. (AVI 5926 kb)



**Additional file 4:** Video extended 24 h. In vivo imaging of monocyte infiltration at 24 h. Extended, wide-field video at 24 h taken in a different mouse than the previous videos. RFP^+^ cells can be seen coursing through large caliber vessels, periodically pausing and adhering to the vessel walls. RFP^+^ cells are also evident in the parenchyma of the central retina. A color-coded time-lapse, where each pixel is colorized based on the time epoch of the video, and a max projection image are given in Fig. 2d. Scale bar is 100 μm. (AVI 36713 kb)



**Additional file 5:** Video 48 h. In vivo imaging of monocyte infiltration at 48 h. By 48 h, RFP^+^ cells had escaped the vasculature and appeared slowly moving throughout the retina. In addition, rapidly moving RFP^+^ cells are evident coursing through the vasculature more quickly than previously observed at 24 h. Recorded from the same vessels shown at 0 h and 24 h. Cells were pseudocolored red; scale bar given in Fig. 2c. (AVI 7900 kb)


### Infiltrating cells are recruited from retinal, not choroidal, vasculature

**Fig. 3:**
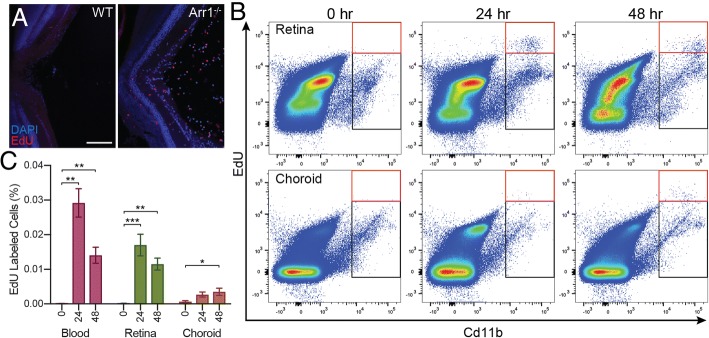
*Infiltrating cells are recruited from the retinal vasculature.* To determine whether leukocytes were primarily infiltrating through the retinal vasculature or the choroidal vasculature, IP injections of EdU were performed at light onset to label dividing cells. **a** IHC of a section through the optic nerve head of WT and *Arr1*^*−/−*^ retinas at 24 h showed EdU staining (red) predominantly of infiltrating cells at the vitreoretinal surface of *Arr1*^*−/−*^ mice. Scale bar 100 μm. Similar results were observed in 2–3 sections from 2 to 4 mice of both genotypes. **b** Flow cytometry was used to quantify CD11b^+^ EdU^+^ cells (red box) from retina, choroid, and blood of *Arr1*^*−/−*^ mice. Black box indicates the number of CD11b^+^ not labeled with EdU. **c** CD11b^+^ EdU^+^ labeled cells were abundant in the retina at 24 and 48 h. In contrast, CD11b^+^ EdU^+^ cells in the choroid did not significantly increase until 48 h and even then were far more rare than in blood and retina. Blood samples were used to verify EdU incorporation and, as expected, showed a significant increase at 24 and 48 h. *p < 0.05, **p < 0.01, ***p < 0.001; error bars are SEM from 3 mice (blood) or 6 retina/choroid per time point

Since photoreceptors are located closer to the choroidal vasculature than the vessels at the vitreoretinal surface, we examined the possibility that monocytes were also entering from the choroidal direction, across Bruch’s membrane, disrupting the retinal pigment epithelium (RPE). This would be difficult to detect by SLO and OCT because of the intrinsic light scattering by the melanin in the RPE and difficult to detect by standard immunohistochemistry (IHC). To determine where cells were infiltrating from, intraperitoneal injections of EdU were performed at light onset to label dividing cells, such as leuokocytes in the bloodstream. Retinal sections stained for EdU after 24 h of light exposure showed a high density of EdU^+^ cells around the optic nerve head and within the adjacent vitreous but not in the outer retinal layers or choroid of *Arr1*^*−/−*^ mice (Fig. 3a). No EdU^+^ cells were evident in dark-reared wildtype (WT) mice treated in parallel, consistent with no cell division or infiltration in healthy retina. EdU pulses initiated at later time points (e.g., 24 or 48 h) incorporated into circulating leukocytes but did not appear in the retina (*data not shown*), supporting the existence of a short, transient period of extravasation evident by IHC and in vivo imaging (Figs. 1, 2, and 3).

To quantify EdU^+^ cells in *Arr1*^*−/−*^ mice, we used flow cytometry on dissociated retina, choroid, and blood samples at light onset (0 h) and afterwards (24, 48 h) (Fig. 3b, c). Negligible numbers of EdU^+^ cells were detected initially (0 h) since EdU would not have had time to incorporate into newly synthesized DNA. By 24 h, there were abundant EdU^+^ cells in the blood, consistent with normal leukocyte proliferation. Notably, EdU^+^ cells were also abundant in the retina at 24 h, but not in the choroid, indicating that EdU^+^ cells from the bloodstream were not entering the retina through the choroidal compartment. In both blood and retinal samples, fewer EdU^+^ cells were measured at 48 h, presumably because the population of labeled cells was turning over (Fig. 3c). These data demonstrate that infiltrating cells rapidly enter through the retinal vasculature as opposed to the choroidal vasculature within 24 h of neurodegeneration onset (Fig. 1b) (see also [24]).

### CD45^high^ cells rapidly increase in the retina during early neurodegeneration

To identify the specific classes of cells infiltrating the retina, we used a flow cytometry gating strategy similar to those described in O’Koren et al. [9] on samples of retina, choroid, and blood from *Arr1*^*−/−*^ mice exposed to 0, 24, 48, 72, or 96 h of light. Cells trapped within the vasculature at the time of dissection were labeled with a fluorescently tagged antibody to CD45 (leukocyte common antigen) injected intravenously just before euthanasia. We will refer to the “In Vasculature” population stained by the intravenously delivered CD45 antibody as “*CD45-IV*” to distinguish it from CD45^+^ cells outside of the vasculature, which were labeled subsequently with a separate fluorescently tagged antibody after dissociation.

**Fig. 4:**
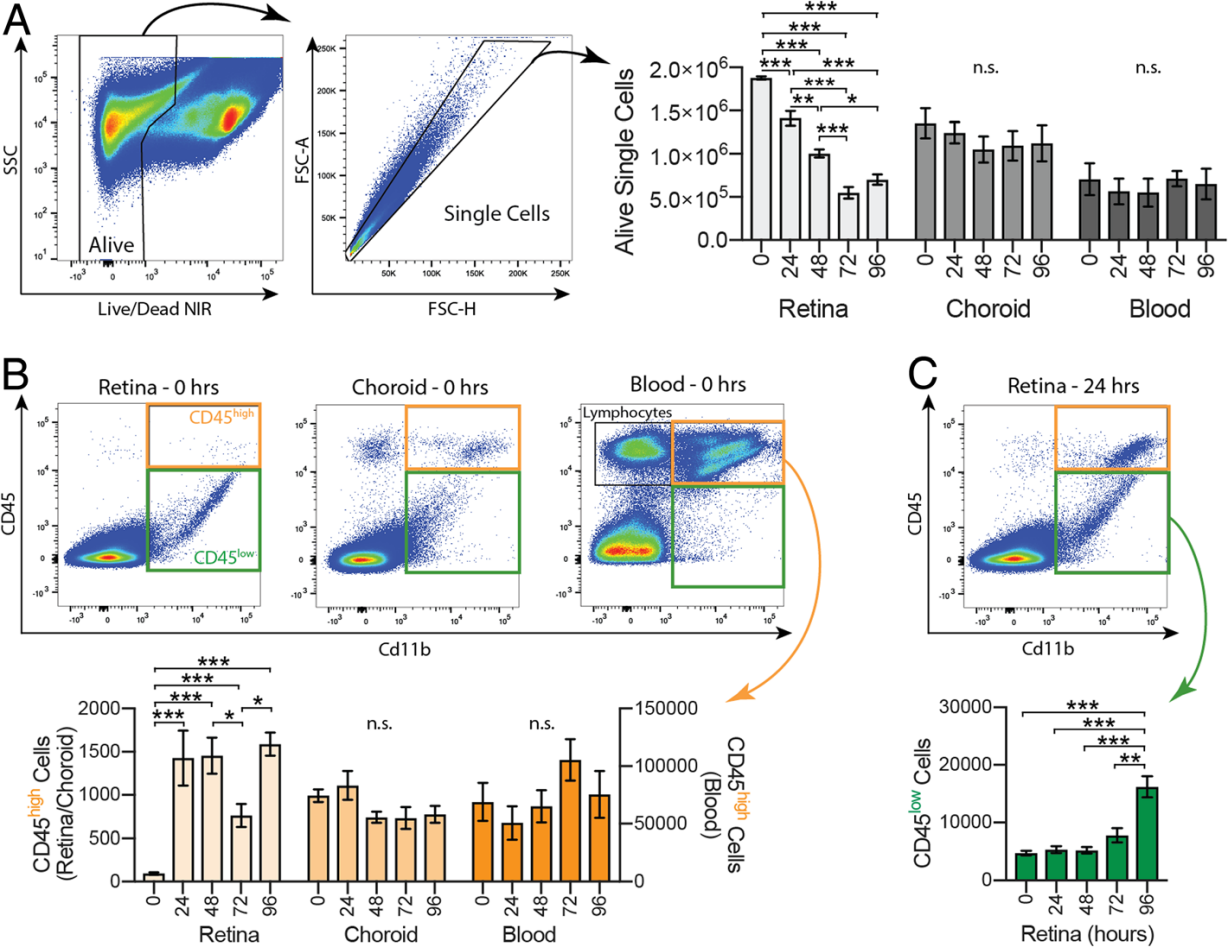
*Increased Cd11b*^*+*^ *CD45*^*high*^ *cells during early neurodegeneration.* Flow cytometry was used to identify cell types recruited during retinal degeneration. **a** Dissociated cell suspensions were gated to select alive, single cells. There was a significant decrease in the number of alive, single cells in the retinal samples at all time points except between 72 and 96 h (light gray), but no significant difference in the choroid (medium gray) or blood (dark gray) samples. **b** Cd11b^+^ cells were gated into CD45^high^ (orange) and CD45^low^ (green) populations. There were very few Cd11b^+^ CD45^high^ cells in the retina at 0 h (light orange); however, by 24 h the number increased dramatically and remained well above baseline at 96 h. There were no significant changes in the choroid (medium orange) or blood (dark orange). **c** The number of retinal Cd11b^+^ CD45^low^ cells (green) remained constant through the first 72 h, then showed a significant population increase at 96 h. *p < 0.05, **p < 0.01, ***p < 0.001; n.s. = not significant; error bars are SEM from 3 to 5 mice (blood) or 6–9 retinas/choroids per time point

Samples were gated by SSC, FSC, and Live/Dead fluorescence to identify alive, single cells (Fig. 4a, scatter plots). There was a progressive decrease in the number of alive, single cells in the retinal samples after light onset, reflecting the on-going photoreceptor degeneration (Fig. 4a, light gray), and consistent with the retinal thinning seen by IHC and OCT in vivo imaging. In contrast, there were no significant changes in the number of alive, single cells harvested from choroid or blood over time (Fig. 4a, medium/dark gray).

To isolate and quantify immune cell subtypes, samples were gated by Cd11b and CD45 expression, which included CD45-IV^+^ cells (Fig. 4b). Prior to the onset of degeneration (*t* = 0), there were very few Cd11b^+^ CD45^high^ cells in the retina, but by 24 h, the number had increased dramatically and remained well above baseline even after 96 h (Fig. 4b, c, light orange). In contrast, there was a large population of Cd11b^+^ CD45^high^ cells in both the choroid and blood samples prior to the onset of degeneration (Fig. 4b, medium/dark orange) and no significant change in either group at later times, indicating that the pools of immune cells within these compartments were unperturbed by the rapid degeneration occurring on the other side of the blood-retinal barrier. The number of cells in the retinal sample that were Cd11b^+^ CD45^low^, which includes resident retinal immune cells like microglia, remained remarkably constant through the first 72 h, then showed a significant population increase at 96 h (Fig. 4c, green).

### Resident immune cell populations are stable during early retinal degeneration

**Fig. 5:**
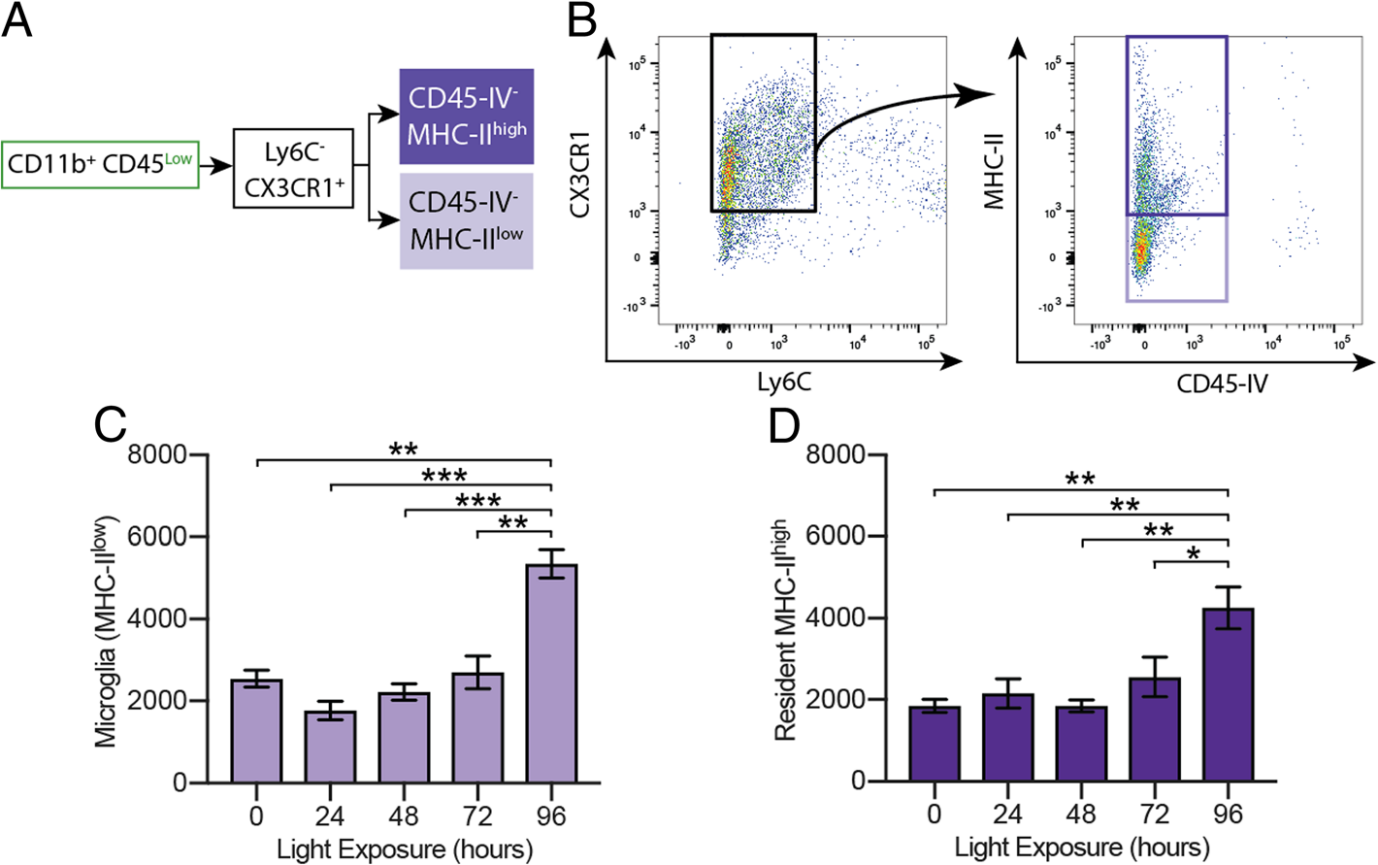
*Microglia numbers are stable during a dramatic period of cell clearance.* **a**, **b** CD45^low^ populations were gated on Ly6C and Cx3CR1 expression; MHC-II was used to distinguish microglia from resident macrophages, and CD45-IV was used to remove cells within or associated with the vasculature. **c** Microglia, the resident MHC-II^low^ cells, remained at constant for the first 72 h, then increased at 96 h (lavender). **d** The number of resident MHC-II^high^ cells was also stable until 96 h (dark purple). Together, these data show that the number of resident immune cells is stable during early retinal degeneration. *p < 0.05, **p < 0.01, ***p < 0.001; error bars are SEM, *n* = 6–9 retinas per time point

The constancy of Cd11b^+^ CD45^low^ retinal cell numbers during the early stages of degeneration (Fig. 4c) led us to examine this population further for discernible subpopulations that might be changing concurrently. First, CD45^low^ cells were gated on Ly6C^−^ and Cx3CR1^+^ expression (Fig. 5a, b). Next, cells were gated on Major Histocompatibility Complex class II (MHC-II), which is normally found on antigen-presenting cells such as macrophages, and CD45-IV, which only labeled cells within or associated with the vasculature at the time of tissue harvest. As expected, very few Ly6C^−^ cells were stained by the CD45-IV antibody injection (Fig. 5b, right), consistent with the CD45^low^ Ly6C^−^ cells being a resident population. Cells that were Cd11b^+^ CD45^low^ Ly6C^−^ Cx3CR1^+^ CD45-IV^−^ and MHC-II^low^, which included the vast majority of microglial cells, remained at a constant level for the first 72 h of degeneration, increasing above baseline only at 96 h (Fig. 5c). These data indicate that microglial proliferation did not occur within the first 72 h, despite widespread microglial activation and photoreceptor phagocytosis (Fig. 1). Likewise, the number of cells that were MHC-II^high^, which include macrophages [9] and putative resident dendritic cells [34], was unchanged during early degeneration, increasing only at 96 h (Fig. 5d). Together, these results demonstrate that the number of resident immune cells in the retina is stable throughout early neurodegeneration, even during a dramatic period of neuronal cell death.

### Infiltrating cells are primarily CD45^high^ Ly6C^high^ monocytes

**Fig. 6:**
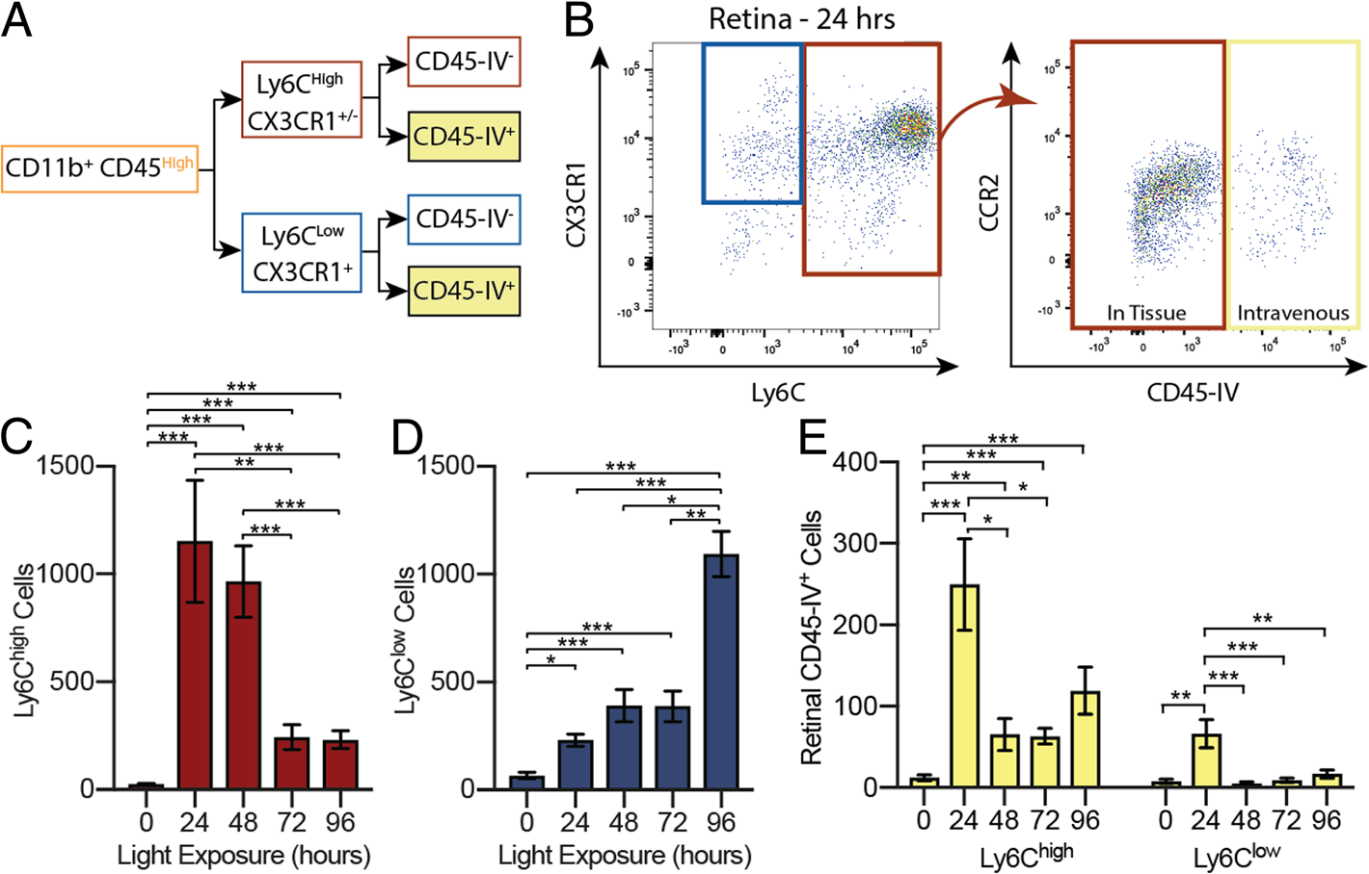
*Infiltrating cells are primarily CCR2*^*+*^ *Ly6C*^*high*^*.* **a**, **b** CD45^high^ populations were gated on Ly6C, Cx3CR1, CCR2, and CD45-IV expression. **c** The number of Ly6C^high^ cells increased dramatically at 24 h and remained high before falling at 72 and 96 h (red). **d** Ly6C^low^ cells were less abundant at early times, slowly increasing over the first 48 h before abruptly doubling between 72 and 96 h (blue). **e** CD45-IV^+^ populations from retinal samples were examined to determine the Ly6C status of cells trapped within the retinal vasculature (yellow). The number of Ly6C^high^ cells increased sharply by 24 h and did not return to baseline by 96 h; the number of Ly6C^low^ cells increased briefly at 24 h before returning to baseline. *p < 0.05, **p < 0.01, ***p < 0.001; error bars are SEM, *n* = 6–9 retinas per time point

To define the molecular phenotypes of infiltrating cells, retinal Cd11b^+^ CD45^high^ cells (Fig. 4b, light orange) were gated on Ly6C, Cx3CR1, CCR2, and CD45-IV expression (Fig. 6a, b). The number of Cd11b^+^ CD45^high^ CD45-IV^−^ Ly6C^high^ cells, which include classical monocytes, increased dramatically by 24 h and remained high before falling at 72 h (Fig. 6c, red). In contrast Cd11b^+^ CD45^high^ CD45-IV^−^ Ly6C^low^ cells were far less abundant at early times, slowly increasing over the first 48 h before abruptly doubling between 72 and 96 h (Fig. 6d blue). Because there was not a corresponding, preceding abrupt increase in Ly6C^high^ cells at 72 h (Fig. 6c), the doubling of Ly6C^low^ cells likely reflects proliferation of infiltrated cells between 72 and 96 h, coincident with the ~ 2-fold increase of CD45^low^ resident cells observed at this time (Fig. 5c, d).

Notably, the population of Ly6C^high^ cells in the retina declined as the population of Ly6C^low^ cells increased (compare Fig. 6c, d), suggesting that Ly6C^high^ cells downregulated Ly6C as they transformed upon entering the retina. To see whether Ly6C^low^ cells were arising from in-residence transformation rather than recruitment, we examined the Ly6C status of monocytes trapped within the retinal vessels at the time of tissue harvest. Because cells adherent to the vessels were stained by CD45-IV, we quantified the number of CD45-IV^+^ cells within the Ly6C^high^ and Ly6C^low^ subpopulations (Fig. 6a, b, yellow boxes). After 24 h, there was a sharp increase in the number of CD45-IV^+^ Ly6C^high^ cells in the retinal samples (Fig. 6e). The number of CD45-IV^+^ Ly6C^high^ cells decreased dramatically between 24 and 48 h, but had not returned to baseline by 96 h; in contrast, the number of CD45-IV^+^ Ly6C^low^ cells increased only slightly at 24 h then dropped back to baseline. The total number of Ly6C^high^ and Ly6C^low^ cells in the blood did not change over time (*p* = 0.5572, *p* = 0.3381, respectively). These results demonstrate that Ly6C^high^ circulating immune cells were preferentially adhering to the retinal vasculature throughout degeneration, but particularly at 24 h, while few Ly6C^low^ cells adhered only briefly at 24 h. This further supports the idea that the late-appearing Ly6C^low^ population (Fig. 6d) arose from transformation of the Ly6C^high^ population within the retina.

### Circulating immune cells are recruited via CCL2-CCR2-mediated signaling

**Fig. 7:**
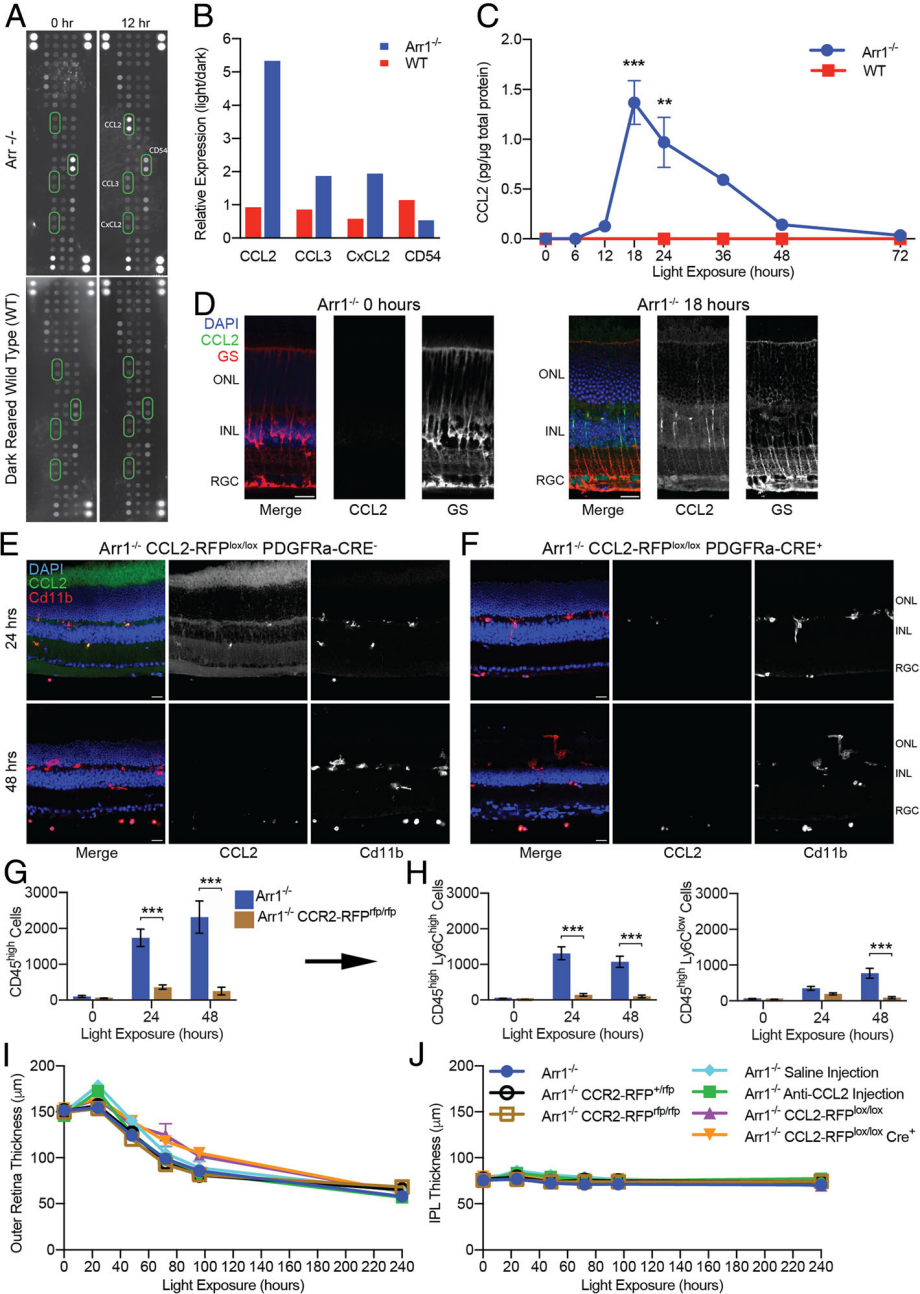
*CCL2-CCR2 mediated signaling recruits monocytes but does not limit the rate of retinal thinning.* **a**, **b** Cytokine array screening of retinal homogenates of dark-reared *Arr1*^*−/−*^ and WT mice before (0 h) and after 12 h of light exposure. There were few light-dependent changes overall, other than a striking increase in CCL2 expression in *Arr1*^*−/−*^ retinas. Representative results from 8 blots, 4 from each genotype. **c** ELISA analysis of dark-reared *Arr1*^*−/−*^ and WT mice revealed that CCL2 levels rose rapidly after 12 h of light exposure, peaking at 18 h (*n* = 3–5 mice per time point). **d** IHC of retinal sections of dark-reared (0 h) and light-exposed (18 h) *Arr1*^*−/−*^ mice showed early CCL2 expression (green) in Müller glial cells (red). **e** Expression of fluorescently tagged CCL2 (green) was observed abundantly throughout the retina at 24 h but not at 48 h, consistent with the previous IHC and ELISA data. **f** In PDGFRa-Cre^+^ mice, the LoxP sites flanking CCL2 were excised only in Müller glia cells, preventing these cells from producing CCL2, and resulting in an almost complete absence of CCL2 in the retina of Cre^+^ mice at 24 h. Only punctate staining was observed in circulating and infiltrating monocytes, which did not lose their ability to produce CCL2. Representative images from 2 to 3 sections from 2 mice per strain per time point. These data demonstrate that Müller glia cells are the primary producers of CCL2 in the retina. **g** Knocking out CCR2, the primary receptor for CCL2, resulted in an almost complete loss of recruited CD45^high^ cells. **h** There was a significant loss of both CD45^high^ Ly6C^high^ and CD45^high^ Ly6C^low^ subpopulations (*n* = 4–6 retinas per strain per time point). **i**, **j** In vivo OCT B-scans were used to measure the thickness of the outer and inner retinal layers during degeneration. **i** Blocking CCL2-CCR2 signaling did not affect the extent of outer retinal thinning in CCR2-RFP^rfp/rfp^ knockout mice (compare *Arr1*^*−/−*^ (blue), *Arr1*^*−/−*^ *CCR2-RFP*^*+/rfp*^ (black), and *Arr1*^*−/−*^ *CCR2-RFP*^*rfp/rfp*^ (brown)), by using an intravitreal injection of CCL2 neutralizing antibody (compare saline (light blue) to anti-CCL2 (green)), or with a conditional CCL2 knockout in Müller glia (compare *Arr1*^*−/−*^ *CCL2-RFP*^*lox/lox*^ *PDGFRa-Cre*^*−*^ (purple) to *Arr1*^*−/−*^ *CCL2-RFP*^*lox/lox*^ *PDGFRa-Cre*^*+*^ (orange)). **j** No appreciable change was detected in the thickness of the inner plexiform layer (IPL) in any of the mice, confirming that retinal degeneration was limited to the photoreceptor layer (*n* = 3–4 retinas from 2 mice per strain per time point). Error bars represent SEM; **p < 0.01, ***p < 0.001; ONL outer nuclear layer, INL inner nuclear layer, RGC retinal ganglion cell layer. Scale bar is 20 μm in **d**–**f**

To begin to identify the signaling mechanism(s) that attract microglia and monocytes to the ONL, we used a 40-chemokine array to screen changes in retinal cytokine expression. After 12 h of light exposure, only four cytokines showed any appreciable change in *Arr1*^*−/−*^ mice. CCL2, previously Monocyte Chemoattractant Protein (MCP-1), changed the most dramatically, increasing approximately 4-fold (Fig. 7a, b; Additional file 6: Table S1). Two other cytokines involved in polymorphonuclear leukocyte recruitment, namely CCL3 and CXCL2, also showed a slight increase in expression. CCL3, previously Macrophage Inflammatory Protein 1-alpha (MIP-1α), is involved in inflammatory recruitment and activation of granulocytes, and CXCL2, previously Macrophage Inflammatory Protein 2-alpha (MIP-2), is a chemoattractant secreted by monocytes and macrophages. The only cytokine that decreased at 12 h in *Arr1*^*−/−*^ mice was CD54, previously Intercellular Adhesion Molecule-1 (ICAM-1), which is a cell adhesion molecule present on the surface of endothelial cells and leukocytes involved in transendothelial migration.

Since CCL2 showed the most substantial change, we used ELISAs to validate the chemokine array and to quantify CCL2 levels in the retina over the course of degeneration. CCL2 expression peaked 18 h after light onset and slowly returned to baseline by 72 h (Fig. 7c), despite continued light exposure and on-going retinal thinning. In contrast, dark-reared WT mice exposed to light in parallel showed no change in cytokine expression in either the cytokine array or ELISAs (Fig. 7a–c; Additional file 6: Table S1).

To determine the source CCL2, we performed IHC on *Arr1*^*−/−*^ retinas. Staining for CCL2 was undetectable in dark-reared *Arr1*^*−/−*^ retinas, but diffuse staining appeared after 18 h of light exposure, particularly in the inner nuclear and retinal ganglion cell layers (Fig. 7d, green). Particularly strong CCL2 staining was evident in individual cells that resembled Müller glia cells (Fig. 7d, red), consistent with other models of retinal degeneration [35].

Next, we used a conditional ablation strategy to completely eliminate Müller glia-derived CCL2 [36]. *Arr1*^*−/−*^ *CCL2-RFP*^*lox/lox*^ mice expressing a fluorescently tagged CCL2 and flanked by loxP sites were crossed with a mouse containing Cre recombinase targeted to Müller glia cells (*Arr1*^*−/−*^ *CCL2-RFP*^*lox/lox*^ *PDGFRa-Cre*^*+*^ mice). After 24 h of light exposure, *Arr1*^*−/−*^ *CCL2-RFP*^*lox/lox*^ *PDGFRa-Cre*^*−*^ mice showed widespread CCL2 staining throughout the retina (Fig. 7e, green), whereas *Arr1*^*−/−*^ *CCL2-RFP*^*lox/lox*^ *PDGFRa-Cre*^*+*^ mice showed almost no detectable CCL2 within the retinal layers; only a small amount of punctate CCL2 staining remained. This punctate staining was also Cd11b^+^, consistent with these cells being a small number of remaining infiltrating cells (Fig. 7f, green). Further, the absence of detectable CCL2 staining in Müller cell-specific CCL2 knockout retinas indicates that RPE-derived CCL2 did not measurably contribute to retinal CCL2 levels. By 48 h, retinal CCL2 expression had dramatically decreased in Cre-negative mice (Fig. 7e, f, bottom panels), consistent with the transient CCL2 expression quantified by ELISA (Fig. 7c). Together, these data show that Müller glia transiently express CCL2 during the initial phase of the retinal degeneration and further reveal them to be the predominant source of retinal CCL2.

To test whether circulating immune cells were being recruited through CCL2-CCR2 mediated signaling, we used *Arr1*^*−/−*^ *CCR2*^*rfp/rfp*^ knock out mice that expressed RFP in lieu of functional CCR2 receptors. Infiltrating cells were quantified using a modified flow cytometry panel that accounted for the RFP fluorescence and gated as previously described. Removing CCR2-mediated signaling caused an almost complete loss of CD11b^+^ CD45^high^ cells recruited to the retina in *Arr1*^*−/−*^ *CCR2*^*rfp/rfp*^ mice at 24 and 48 h (Fig. 7g). This was true of both Ly6C^high^ and Ly6C^low^ subpopulations (Fig. 7h). In addition, using immunohistochemistry, we quantified the density of CD11b^+^ cells within the retina of the Müller cell-specific CCL2 knockout (*Arr1*^*−/−*^ *CCL2-RFP*^*lox/lox*^ *PDGFRa-Cre*^*+*^ vs *Arr1*^*−/−*^ *CCL2-RFP*^*lox/lox*^ *PDGFRa-Cre*^*−*^; Fig. 7e, f). At 48 h after the onset of degeneration, the density of Cd11b + cells was significantly reduced (Cre^−^ 1.20 × 10^−4^ cells/μm^2^ +/− 1.8 × 10^−6^ vs. Cre^+^ 9.03 × 10^−5^ cells/μm^2^ +/− 4.8 × 10^−6^; *p* = 0.0097; *n* = 4 sections from 2 eyes per strain), indicating that blocking Müller cell CCL2 expression also reduced monocyte infiltration. Together, these data show that monocytes are recruited during neurodegeneration through a CCR2-mediated signaling pathway, likely in direct response to the release of CCL2 by Müller glia cells.

### Eliminating CCL2-CCR2 signaling did not alter the rate of retinal thinning

To determine the effect of eliminating CCL2-CCR2 mediated recruitment, we measured the rate of retinal thinning in *Arr1*^*−/−*^ *CCR2*^*rfp/rfp*^ mice using in vivo OCT imaging. Surprisingly, the rate of thinning did not significantly change in mice lacking functional CCR2 receptors. Instead the photoreceptor layer degenerated at the same rate in *Arr1*^*−/−*^ mice (Fig. 7i, dark blue), *Arr1*^*−/−*^ *CCR2*^*+/rfp*^ heterozygotes (black), and *Arr1*^*−/−*^ *CCR2*^*rfp/rfp*^ homozygous knockouts (brown). These data indicate that blocking the infiltration of monocytes had no effect on the rate of neurodegeneration.

Next, we examined the consequence of blocking CCL2 directly using two different strategies. First, we injected a neutralizing antibody intravitreally during the rising phase of CCL2 expression to block CCL2 function. IHC confirmed that a single injection of CCL2-neutralizing antibody remained in the eye for at least 12 h (Additional file 7: Figure S2). Despite the blockade, in vivo OCT imaging found no significant effect on the overall rate of retinal thinning (Fig. 7i, j; compare saline injected (light blue) vs anti-CCL2 injected (green).

Finally, we used conditional CCL2 ablation mice (*Arr1*^*−/−*^ *CCL2-RFP*^*lox/lox*^ *PDGFRa-Cre*^*+*^*)* to completely eliminate Müller glia-derived CCL2 (Fig. 7e, f). In vivo OCT imaging detected no change in the rate of retinal thinning between *Arr1*^*−/−*^ *CCL2-RFP*^*lox/lox*^ *PDGFRa-Cre*^*−*^ and *Arr1*^*−/−*^ *CCL2-RFP*^*lox/lox*^ *PDGFRa-Cre*^*+*^ mice (Fig. 7i; compare lox/lox Cre^−^ (purple) to lox/lox Cre^+^ (orange)); the photoreceptor layer degenerated at the same rate in CCL2-deficient mice as in experimentally matched controls, while the inner plexiform layer thickness did not change (Fig. 7i, j).

Thus, despite the large, transient increase in CCL2 expression by Müller cells and evidence that blocking the CCL2-CCR2 signaling reduced the influx of CD45^high^ cells, neurodegeneration proceeded unabated. Unlike other retinal damage studies in which monocytes exacerbate degeneration [14, 37, 38], our results demonstrate that monocyte infiltration does not necessarily further harm or control the rate of neurodegeneration.

## Discussion

We have shown that during the onset of retinal degeneration, there are two distinct and simultaneous immune responses: (1) the *en mass* activation and migration of microglial cells to the degenerating neurons (Fig. 1) (see also [24]) and (2) a transient period of peripheral cell infiltration through the retinal vasculature, primarily of classical CD45^high^ Ly6C^+^ monocytes (Figs. 2, 3, 4, and 6). Blocking CCL2-CCR2 signaling disrupts the recruitment of monocytes, but does not rescue the phenotype or change the rate of neurodegeneration (Fig. 7). Thus, the emergent picture of neuroinflammation during acute degeneration is one of marked heterogeneity, involving the interplay of microglia, glia, and infiltrating monocytes throughout the course of disease progression. Here, we discuss our findings in the context of CCL2-CCR2-mediated recruitment by Müller glia, the extravasation of monocytes through the retinal vasculature, and the role of recruited immune cells during neuronal death, with an emphasis on the implications of our results for other forms of neurodegenerative disease.

### CCL2 as a glial-mediated signaling molecule for neuroinflammation

Arrestin-1 is a photoreceptor-specific protein responsible for deactivating light-driven rhodopsin activity [18]. The deletion of Arrestin-1 causes widespread photoreceptor degeneration [21] and synchronized, rapid morphological transformation of retinal microglia [24]. The signal(s) inducing microglial migration to the photoreceptors are not yet known. Here, cytokine arrays, ELISAs, IHC, and conditional knockout of retinal CCL2 show that CCL2 is rapidly expressed by Müller cells, the radial glia of the retina. Although CCL2 staining was observed in the photoreceptor layer in *Arr1*^*−/-*^*CCL2-RFP*^*lox/lox*^ *PDGFRa-Cre*^*−*^ mice (Fig. 7e), consistent with previous studies [39], when Müller glia cells lost their ability to produce CCL2 (in *PDGFRa-Cre*^*+*^ mice), this staining disappeared (Fig. 7f). This indicates that Müller cell-derived CCL2 diffuses throughout the neural retina and that photoreceptors, retinal pigment epithelium (RPE), and microglia are not the predominant sources of CCL2 in the retina at the onset of degeneration.

Although neuroinflammation is typically thought to result from the interaction of microglia and monocytes with dying neurons, our work shows that radial glial cells can act as conduits for relaying stress and death signals beyond the site of degeneration. Müller radial glia are perfectly poised to detect physiological changes accompanying photoreceptor stress through their intimate and numerous contacts with the photoreceptor inner segments and extensive contacts with retinal vessels, which help create the vitreoretinal barrier known as the inner limiting membrane (reviewed in [40]). Therefore, Müller cells, like microglia, can be considered “first responders” to neuronal injury and degeneration.

Concurrent with the peak expression of CCL2 in Müller cells, our in vivo SLO imaging of *Arr1*^*−/−*^ *CCR2*^*+/rfp*^ mice show that CCR2^+^ monocytes adhere to the endothelial cells of retinal vessels within 24 h of light onset (Fig. 2, Additional file 2: Video 0 h; Additional file 3: Video 24 h; Additional file 4: Video ext 24 h; Additional file 5: Video 48 h). As the rate of degeneration slowed over 48–72 h (Fig. 4a, light gray bars; Fig. 7i), the level of CCL2 returned to baseline and the number of infiltrating CD45^high^ Ly6C^high^ cells decreased (Fig. 6). Eliminating CCL2-CCR2 signaling using *Arr1*^*−/−*^ *CCR2*^*rfp/rfp*^ mice resulted in a significant drop in the recruitment of CD45^high^ monocytes from the blood stream (Fig. 7g), confirming that signaling through the CCL2-CCR2 pathway was responsible for recruitment, consistent with other models [37]. Together these data demonstrate that Müller glia cells respond to retinal neurodegeneration by releasing CCL2, which recruits circulating immune cells to the site of degeneration through the CCL2-CCR2 signaling pathway and that disrupting this pathway prevents the recruitment of monocytes.

### Early response to neurodegeneration dominated by CD45^high^ Ly6C^high^ monocytes

Contrary to expectations, the CD45^high^ Ly6C^high^ infiltrating cells did not enter through the choroid, but rather through the retinal vasculature and optic nerve head (Figs. 2 and 3, Additional file 1: Figure S1), despite the fact that the degeneration was happening in the deeper photoreceptor layers of the retina. This is similar to the way that monocytes enter the eye through the optic nerve head during retinal ganglion cell degeneration in a slower model of neurodegeneration of early glaucoma [41].

Interestingly, the number of resident immune cells in the retina (Cd11b^+^ CD45^low^ Ly6C^−^ Cx3CR1^+^) was remarkably constant for the first 3 days of degeneration, a time during which approximately 40% of the outer retina thickness disappeared (Figs. 1a and 7i) and monocytes invaded the retina. The resident immune cell population consisted of two subpopulations: MHC-II^low^, composed primarily of microglia, and MHC-II^high^, which includes perivascular macrophages and a small, robust population of dendritic cells [34, 42] or a long-lived population of putative macrophages that express high levels of MHC-II [9]. Thus, if the monocytes that infiltrated at 24 h rapidly transformed into macrophages (MHC-II^high^ cells) or microglia-like cells (MHC-II^low^), they either must have replaced cells that were dying or kept high levels of CD45 expression. Further, it was not until between 72 and 96 h after degeneration onset that the total number of CD45^low^ cells substantially increased (and roughly doubled), suggesting a distinct, late phase of cell proliferation in both MHC-II^low^ and MHC-II^high^ subclasses (Fig. 5c, d). Thus, although microglia were the first to migrate to the site of retinal injury, their population did not increase in size until neurodegeneration had proceeded for 96 h. Instead, the dominant responders were the peripheral monocytes, which began infiltrating within 24 h.

Monocytes were abundant in the retina at 24 and 48 h (Fig. 6c). Many of the newly extravasated cells expressed CCR2, but the population was heterogeneous (Fig. 6b). By 72 h, there were roughly twice as many Ly6C^−^ cells than Ly6C^+^, and by 96 h, the discrepancy was more than 4-fold (Fig. 6d). This is consistent with other models of neurodegeneration that have shown Ly6C^+^ classical monocytes, and inflammatory monocytes can infiltrate tissue and differentiate into pro-inflammatory macrophages and dendritic cells ([43, 44] for review). The disproportioned jump in the Ly6C^low^ population at 96 h is similar to the increase observed in the resident population, suggesting that the infiltrating and resident populations proliferated in synchrony. Together, these data demonstrate that the identities of immune cells in the degenerating retina become highly intertwined and heterogeneous once degeneration is initiated, both in terms of lineage (yolk sac-derived microglia vs bone marrow-derived monocytes) and tissue-specific transcriptional regulation [45].

### Neurodegeneration proceeds independent of monocyte recruitment

Despite acting as the dominant responder, the elimination of monocytes did not affect the duration of neurodegeneration. We employed three different experimental techniques to eliminate CCL2-CCR2 signaling, yet despite evidence that infiltration was blocked (Fig. 7g), the photoreceptor layer degenerated at the same rate in CCL2-deficient mice and CCR2-deficient mice as in experimentally matched controls (Fig. 7i), indicating that the influx of monocytes had no effect on the outcome of neurodegeneration.

One possible reason that blocking CCL2-CCR2 signaling in *Arr1*^*−/−*^ did not alter neurodegeneration may be because the rate of degeneration is intrinsic to the mechanism of cell death within the photoreceptors rather than driven by the immune response. The loss of Arrestin-1 only affects photoreceptors, rendering them more than 200-fold more sensitive to light than normal [18] and causing them to die even in dim light (25 lx, without dilated pupils) [24]. The degeneration is a cell-autonomous process, initiated all at once by light onset. This differs from diseases that are slowly progressive, like retinitis pigmentosa or age-related macular degeneration, in which photoreceptor stress is cumulative and may be more sensitive to immune system modulation.

Another fundamental difference between the *Arr1*^*−/−*^ model and studies that have implicated CCL2-CCR2 as an important mediator of retinal health is the degree to which the damage is limited to a single cell type. The loss of photoreceptors in *Arr1*^*−/−*^ mice occurs at light levels that do not cause photolytic damage (75–200 lx, undilated pupils of pigmented mice) or stress the regenerative capacity of the RPE, at least at the early times being considered in this study. This is in contrast to studies that inflicted broader damage, such as Rutar and colleagues [37], who suppressed retinal CCL2 levels with small interfering RNA (siRNA) and found a reduced the number of apoptotic photoreceptors induced by bright light (1000 lx, dilated pupils); Sennlaub and colleagues [14] who knocked out CCL2 and rescued photoreceptor degeneration caused by light stress (4500 lx, dilated pupils); or Hu and colleagues [38] who knocked out CCR2 and found significant structural and functional preservation of the retina after exposure to blue light-induced damage (500 lx, daily for 3 days). The exclusive damage to photoreceptors is presumably why CCL2 is one of only a handful of cytokines that change in the *Arr1*^*−/−*^ at early times (Fig. 7a–c; Additional file 6: Table S1), rather than the noted upregulation of a broad spectrum of chemokines in other studies (e.g., CCL3, CCL4, CCL7, CXCL1, and CXCL10) [46, 47].

Given the interest in developing therapies that target cytokine cascades during retinal disease, the specificity of the *Arr1*^*−/−*^ model provides a notable opportunity to examine the consequences of immune system intervention in a targeted, cell-autonomous model of neurodegeneration. This is critical for determining the effects of cytokine interventions on cell type-specific genetic disorders, for understanding the mechanism(s) by which peripheral cells invade the CNS during disease progression, and for interpreting the consequences of these cells on disease progression during neurodegeneration, particularly if modulation of the innate immune system is to be a viable therapeutic target for neurodegenerative disease (e.g., [48, 49]).

## Conclusions

Here, we show that in the *Arr1*^*−/−*^ retina, the induction of photoreceptor degeneration causes a transient infiltration of monocytes from the retinal vasculature within 24 h. Recruitment occurs after the increase in retinal CCL2 and several days before the resident microglial cells show any change in population size. Microglia and monocyte proliferation occur concurrently, but not until several days after the onset of neurodegeneration, when the rate of cell death has slowed. Eliminating CCL2-CCR2 signaling blocked monocyte infiltration but did not alter the extent of degeneration. These results demonstrate that the immune response to neurodegeneration includes both resident and infiltrated cells, even at very early times and that monocyte involvement is not limited to disease states that overwhelm or deplete the resident microglial population and does not always hasten degeneration.

## Additional files

Additional file 1: **Figure S1.** Monocyte infiltration occurs through retinal vasculature and the optic nerve head. (A) Low and high magnification views of retinal IHC sections from *Arr1*^*−/*−^ mice before (0 h) and after 24 h of light exposure. Note the appearance of small round cells along the vitreal surface after 24 h. Scale bar 200 μm. (B) IHC from sections through the optic nerve head (ONH) of *Arr1*^*−/*−^ retina. Infiltrating cells, many of which were Cd11b^+^ (red), clustered around the ONH and optic stalk. Vitreal Cd11b^+^ cells may include a population of hyalocytes. Scale bar 50 μm. (C) In vivo OCT imaging detected infiltrating cells in the vitreous (arrows), near the optic stalk and peripapillary region, in *Arr1*^*−/−*^ but not wildtype mice. Similar results were seen in more than 6 other mice at this time point. (PNG 3160 kb)

Additional file 6: **Table S1.** Changes in chemokine expression after 12 h of light exposure. A 40-chemokine array was used to screen for cytokine expression in degenerating *Arr1*^*−/−*^ retinas after 12 h of light exposure. Very few cytokines showed any appreciable change compared to dark-reared WT controls. The most dramatic was CCL2, which showed a 5.3-fold increase. Relative expression levels were calculated as light-exposure divided by dark reared for each group, then averaged across runs. A value of 1 indicates no change; shades of red are greater than 1.5, and shades of blue are less than 0.6. (DOCX 15 kb)

Additional file 7: **Figure S2.** CCL2-neutralizing antibody remains in the eye for at least 12 h. A single injection of CCL2-neutralizing antibody 12 h after light onset was used to block CCL2 signaling intravitreally. Flat-mounted retinae were stained with a fluorescently tagged secondary antibody (green) capable of binding to intravitreally injected CCL2 antibody after 1 (B) or 12 (C) hours from the time of injection, demonstrating that the injected antibody remained in the eye. No staining was seen in the saline-injected control (A), verifying that the secondary did not bind indiscriminately. Scale bar is 500 μm. (PNG 755 kb)

## Abbreviations

IHC: Immunohistochemistry
INL: Inner nuclear layer
IPL: Inner plexiform layer
IS: Photoreceptor inner segment layer
OCT: Optical coherence tomography
ONL: Outer nuclear layer
OPL: Outer plexiform layer
OS: Photoreceptor outer segment layer
PBS: Phosphate-buffered saline
PFA: Paraformaldehyde
RFP: Red fluorescent protein
RGC: Retinal ganglion cell layer
RPE: Retinal pigment epithelium
SEM: Standard error of the mean
SLO: Scanning laser ophthalmoscopy
WT: Dark-reared wildtype
