# Supplementary figures and images for: Monocyte infiltration rather than microglia proliferation dominates the early immune response to rapid photoreceptor degeneration
Source: J Neuroinflammation. 2018 Dec 15;15:344. doi: 10.1186/s12974-018-1365-4 (PMC7659426; doi:10.1186/s12974-018-1365-4)

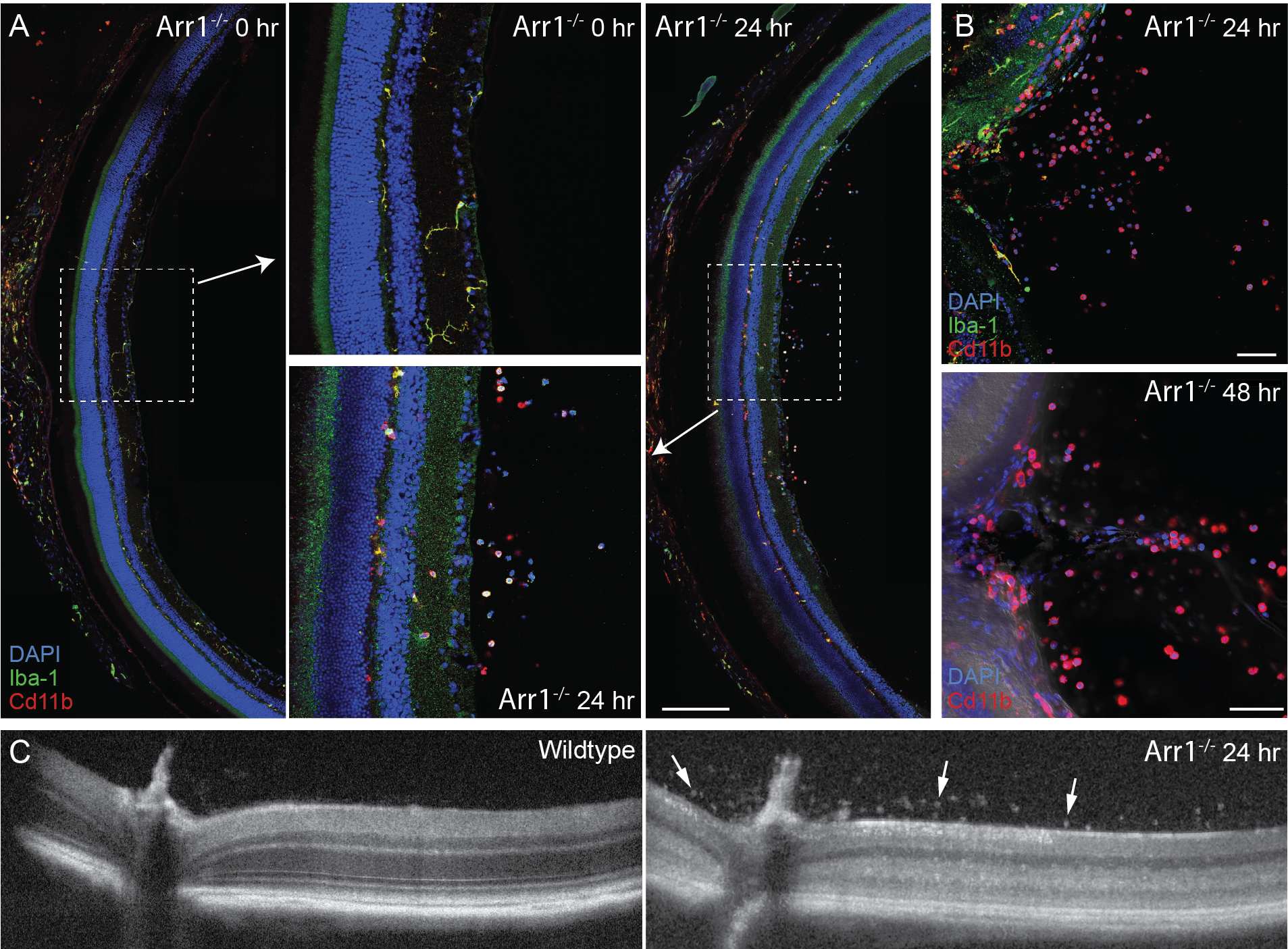

Supplement: Supplementary file 5 — Figure S1. Monocyte infiltration occurs through retinal vasculature and the optic nerve head. (A) Low and high magnification views of retinal IHC sections from Arr1−/− mice before (0 h) and after 24 h of light exposure. Note the appearance of small round cells along the vitreal surface after 24 h. Scale bar 200 μm. (B) IHC from sections through the optic nerve head (ONH) of Arr1−/− retina. Infiltrating cells, many of which were Cd11b+ (red), clustered around the ONH and optic stalk. Vitreal Cd11b+ cells may include a population of hyalocytes. Scale bar 50 μm. (C) In vivo OCT imaging detected infiltrating cells in the vitreous (arrows), near the optic stalk and peripapillary region, in Arr1−/− but not wildtype mice. Similar results were seen in more than 6 other mice at this time point. (PNG 3160 kb) [file 12974_2018_1365_MOESM1_ESM.png]

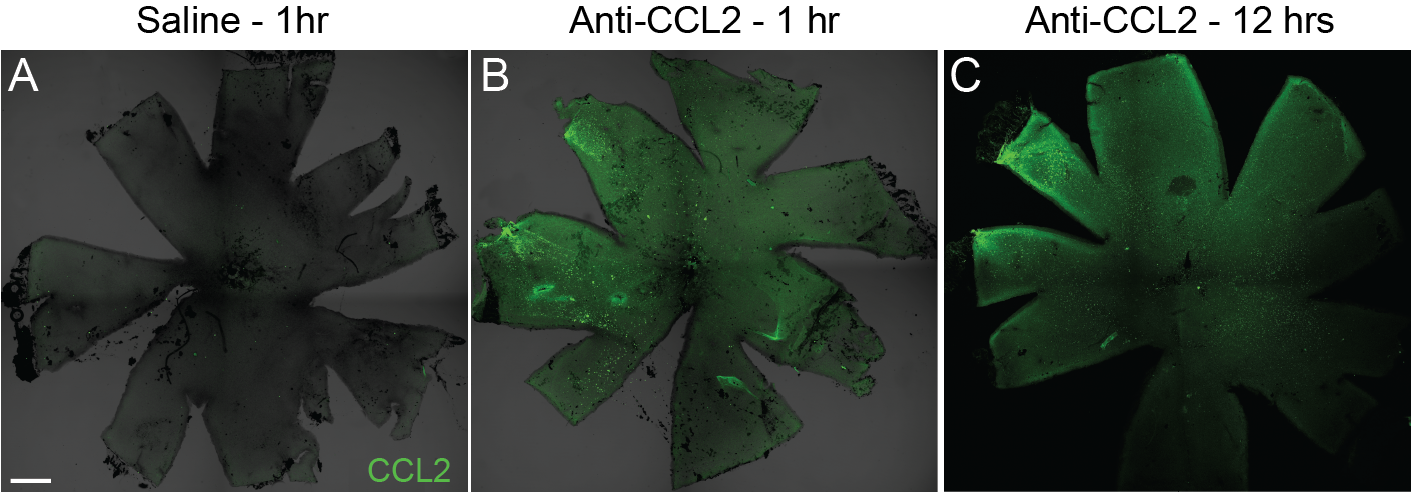

Supplement: Supplementary file 7 — Figure S2. CCL2-neutralizing antibody remains in the eye for at least 12 h. A single injection of CCL2-neutralizing antibody 12 h after light onset was used to block CCL2 signaling intravitreally. Flat-mounted retinae were stained with a fluorescently tagged secondary antibody (green) capable of binding to intravitreally injected CCL2 antibody after 1 (B) or 12 (C) hours from the time of injection, demonstrating that the injected antibody remained in the eye. No staining was seen in the saline-injected control (A), verifying that the secondary did not bind indiscriminately. Scale bar is 500 μm. (PNG 755 kb) [file 12974_2018_1365_MOESM7_ESM.png]
