# Supplementary material for: Monocyte infiltration rather than microglia proliferation dominates the early immune response to rapid photoreceptor degeneration
Source: J Neuroinflammation. 2018 Dec 15;15:344. doi: 10.1186/s12974-018-1365-4 (PMC7659426; doi:10.1186/s12974-018-1365-4)
Supplement: Supplementary file 6 — Table S1. Changes in chemokine expression after 12 h of light exposure. A 40-chemokine array was used to screen for cytokine expression in degenerating Arr1−/− retinas after 12 h of light exposure. Very few cytokines showed any appreciable change compared to dark-reared WT controls. The most dramatic was CCL2, which showed a 5.3-fold increase. Relative expression levels were calculated as light-exposure divided by dark reared for each group, then averaged across runs. A value of 1 indicates no change; shades of red are greater than 1.5, and shades of blue are less than 0.6. (DOCX 15 kb) [file 12974_2018_1365_MOESM6_ESM.docx]

***Table S1 – Changes in chemokine expression after 12 hours of light exposure***

|  | **Dark Reared WT** | ***Arr1^-/-^*** |
| --- | --- | --- |
| C5/C5a | 0.989 | 0.757 |
| CCL1/TCA-3/I-309 | 0.997 | 0.943 |
| CCL2/MCP-1/JE | 0.923 | 5.332 |
| CCL3/MIP-1α | 0.861 | 1.869 |
| CCL4/MIP-1β | 0.970 | 1.021 |
| CCL5/RANTES | 1.272 | 0.941 |
| CCL11/Eotaxin | 0.767 | 1.212 |
| CCL12/MCP-5 | 0.970 | 1.185 |
| CCL17/TARC | 0.835 | 1.018 |
| CD54/sICAM-1 | 1.146 | 0.532 |
| CXCL1/KC | 0.823 | 1.323 |
| CXCL2/MIP-2 | 0.583 | 1.944 |
| CXCL9/MIG | 0.933 | 1.469 |
| CXCL10/CRG-2/IP-10 | 0.830 | 1.018 |
| CXCL11/I-TAC | 0.910 | 0.725 |
| CXCL12/SDF-1 | 0.969 | 1.035 |
| CXCL13/BCA-1/BLC | 0.878 | 0.724 |
| G-CSF | 0.748 | 1.510 |
| GM-CSF | 1.033 | 1.055 |
| IFN-γ | 0.692 | 0.916 |
| IL-1α/IL-1F1 | 0.904 | 1.104 |
| IL-1β/IL-1F2 | 0.637 | 1.288 |
| IL-1ra/IL-1F3 | 0.786 | 0.630 |
| IL-2 | 0.832 | 0.984 |
| IL-3 | 0.971 | 0.840 |
| IL-4 | 0.951 | 0.686 |
| IL-5 | 0.658 | 1.003 |
| IL-6 | 0.869 | 1.075 |
| IL-7 | 0.821 | 0.888 |
| IL-10 | 0.949 | 0.969 |
| IL-12p70 | 1.109 | 0.774 |
| IL-13 | 0.904 | 0.958 |
| IL-16 | 0.819 | 0.991 |
| IL-17 | 0.689 | 1.260 |
| IL-23 | 0.940 | 0.957 |
| IL-27 | 1.153 | 0.747 |
| M-CSF | 0.844 | 1.237 |
| TIMP-1 | 0.858 | 0.967 |
| TNF-α | 0.852 | 1.071 |
| TREM-1 | 0.816 | 0.963 |

A 40 chemokine array was used to screen for cytokine expression in degenerating *Arr1^-/-^* retinas after 12 hours of light exposure. Very few cytokines showed any appreciable change compared to dark reared WT controls. The most dramatic was CCL2, which showed a 5.3 fold increase. Relative expression levels were calculated as light-exposure divided by dark reared for each group, then averaged across runs. A value of 1 indicates no change; shades of red are greater than 1.5, and shades of blue are less than 0.6.
